# High Prevalence of Infectious Diseases and Drug-Resistant Microorganisms in Asylum Seekers Admitted to Hospital; No Carbapenemase Producing Enterobacteriaceae until September 2015

**DOI:** 10.1371/journal.pone.0154791

**Published:** 2016-05-04

**Authors:** Sofanne J. Ravensbergen, Mariëtte Lokate, Darren Cornish, Eveline Kloeze, Alewijn Ott, Alex W. Friedrich, Rob van Hest, Onno W. Akkerman, Wiel C. de Lange, Tjip S. van der Werf, Erik Bathoorn, Ymkje Stienstra

**Affiliations:** 1 Department of Internal Medicine/Infectious Diseases, University of Groningen, University Medical Centre Groningen, Groningen, The Netherlands; 2 Department of Medical Microbiology, University of Groningen, University Medical Centre Groningen, Groningen, The Netherlands; 3 Primary Health Care Centre for Asylum Seekers (Gezondheidscentrum Asielzoekers), Ter Apel, The Netherlands; 4 Department of Medical Microbiology, Certe, Groningen, The Netherlands; 5 Department of Tuberculosis Control, Regional Public Health Service Groningen, Groningen, The Netherlands; 6 Department of Pulmonary Diseases and Tuberculosis, University of Groningen, University Medical Centre Groningen, Groningen, The Netherlands; Health Protection Agency, UNITED KINGDOM

## Abstract

**Introduction:**

The current refugee crisis emphasizes the need for information on infectious diseases and resistant microorganisms in asylum seekers with possible consequences for public health and infection control.

**Methods:**

We collected data from asylum seekers admitted to our university hospital or who presented at the Emergency Department (n = 273). We collected general and demographic characteristics including country of origin, the reason of presentation, and the screening results of multi-drug resistant organisms.

**Results:**

67% of the patients were male with a median age of the study group of 24 years (IQR 15–33); 48% of the patients had an infectious disease—predominantly malaria with *P*. *vivax* or tuberculosis. Patients also reported with diseases which are less common—e.g. leishmaniasis, or even conditions rarely diagnosed in Europe—e.g. louse borne relapsing fever. A carriage rate of 31% for multi-drug resistant microorganisms (MDRO) was observed, with ESBL-expressing *E*.*coli* (n = 20) being the most common MDRO. No carriage of Carbapenemase Producing Enterobacteriaceae was found.

**Conclusion:**

The current refugee crisis in Europe challenges hospitals to quickly identify and respond to communicable diseases and the carriage of MDRO. A rapid response is necessary to optimize the treatment of infectious diseases amongst asylum seekers to maximize infection control.

## Introduction

The current refugee crisis in Europe challenges both society as a whole and health care workers. Six hundred and twenty-six thousand people applied for asylum in the 28 European (EU) Member States in 2014. When compared to 2013, this was an increase of 45%[1]. More than 350,000 refugees reported at the EU borders between January-September 2015. This number may be an underestimate as many refugees may have remained undetected[2, 3].

Next to travelling, migration is a well-known factor in the spread of infectious diseases and multi-drug resistant micro-organisms (MDRO) [4]. However, little is known about the carriage of infectious diseases and MDRO in asylum seekers whenever they report with illness to the healthcare system in the host country with possible implications for hospital infection control.

The Netherlands have a very active surveillance for MDRO with a very low MDRO prevalence among patients admitted to hospital [5]. The overall carriage of extended spectrum beta-lactamase (ESBL) producing bacteria in the Dutch population is 5.1% [6]. In hospitals the overall carriage rate of vancomycin-resistant enterococci (VRE) is 0.4% and the percentage of methicillin-resistant *Staphylococcus aureus* (MRSA) is only 2%. Most cases of Carbapenemase-producing *Enterobactereaceae* (CPE) in the Netherlands have been reported in patients repatriated from a foreign hospital [7], although some hospital outbreaks have occurred[8]. The Netherlands has a strict hospital infection prevention policy, especially with respect to screening for patient admissions for those recently admitted to hospitals in foreign countries. Screening policy does not include travellers and it is currently unclear whether asylum seekers without a recent hospital admission would need to be screened.

In addition to the carriage of MDRO, asylum seekers may present with infectious diseases which may have consequences for public health and hospital hygiene; immigrants are known to have a higher rate of tuberculosis compared to the indigenous population [9,10]. However rates may vary considerably between countries of origin. The incidence of tuberculosis is 78 per 100.000 inhabitants in Eritrea and 17 per 100.000 inhabitants in Syria [11]. Information on MDRO carriage in countries of origin is scarce. In Syria the rate of MDR gram negative bacteria in selected patient populations with clinical infections was around 50–60%. [12, 13].

The spectrum of infectious diseases asylum seekers present with depends on risk factors such as country of origin, exposure during travel, previous living conditions, and access to health care and migration routes [14].

Here we report the spectrum of infectious diseases, prevalence of patients carrying MDRO’s amongst asylum seekers who presented to the University Medical Centre Groningen which is located close to the national registration centre for asylum seekers in the Netherlands. Our data may conceivably help improve adequate care for asylum seekers with infectious diseases and enable optimal hospital hygiene strategies.

## Materials and Methods

### Asylum Seeking Procedure in the Netherlands

In 2014 24,929 asylum seekers arrived in the Netherlands, an increase of almost 62% compared to 2013 (15,394). In the context of the current European refugee crisis, the number of asylum seekers in 2015 has increased considerably. Since January 2015 up until the beginning of September 2015, 33,598 asylum seekers had already reported at the national registration centre [15].

The Netherlands operate a centralised system of asylum application. Apart from a small minority at the national airport Schiphol and unaccompanied minors, the majority of asylum seekers must file their request at the national registration centre in Ter Apel. Within the first three days following arrival individuals are identified, registered and screened for active pulmonary tuberculosis. In Spring 2014 a standard preventative treatment of scabies was introduced. Screening is performed by the municipal health services. All asylum seekers are insured by the same insurance company and have an insurance number that starts with 9010 as decided by the insurance company. After this period, asylum seekers move to one of the asylum centres in the Netherlands to await processing [16].

### Screening at Admission to the Hospital

The University Medical Centre Groningen is the university hospital closest to the national registration centre (60 km) and a preferred carrier for treating infectious diseases. The general practitioner based at the national registration centre decides whether the asylum seeker is referred to the regional hospital or the university hospital and for abnormalities found during TB screening the TB control physician in Groningen decides.

General infection prevention policy in the Netherlands includes screening for MRSA, VRE and resistant gram-negative bacteria of all patients who admitted to a hospital outside the Netherlands in the past 2 months.

In April 2014, the department of medical microbiology in the UMCG advised screening for MRSA, VRE, and multidrug resistant gram negatives for all asylum seekers admitted to the hospital or who presented at the emergency department (with a high probability of a subsequent admission). This advice was only given if admission or outpatient visit was reported. The reason for screening was the anticipated high carriage rate of MDRO in asylum seekers when considering their countries of origin. Asylum seekers who were admitted or presented to the emergency department were screened for carriage of the following MDRO’s: MRSA, ESBL, fluorquinolone- and aminoglycoside-resistant (MDR) Gram-negative bacteria, VRE, and CPE as part of standard care.

Carriage of MDRO does not have consequences for hospital hygiene measure in the outpatient setting. Therefore patients only visiting out-patient departments were not included in the MDRO screening.

### Selection of Participants

A retrospective study was conducted at the UMCG. All asylum seekers admitted to the UMCG or reporting to the emergency department between April 1st 2014 through September 1st 2015 were included. Patients were identified as asylum seekers based on their specific insurance number. Patients with the specific insurance number but whose asylum request was rejected by legal authorities as evidenced by the information available in their medical records were not included. Only asylum seekers who presented at the emergency department or who were admitted to the wards, or the tuberculosis department were included. If patients were admitted more than once, only the first admission in the study period was included.

General characteristics such as age at admission, gender, country of origin, and arrival data in the Netherlands, admission period and reason of admission were collected. Detailed information was collected concerning infectious conditions the patients presented with. The ICD-10 classification was used to describe the non-infectious diseases patients presented with at the hospital [17].

### Screening for MDRO

Screening for MDRO consisted of swabs from nose, throat, rectum and perineum. MRSA was tested on nose, throat and perineum swabs with PCR (GeneXpert Cepheid). These swabs were also cultured on enrichment broth and chromID-MRSA plates (Biomerieux). VRE was detected as described previously [18]. Presence of MDR Gram-negative bacteria in throat and rectum swabs was detected by culture on selective agar plates (3-com Iso sensitest agar ME/CF/CX and CI/TO/PT, Mediaproducts, Groningen, the Netherlands). Antibiotic susceptibility was tested by automated susceptibility testing (VITEK2, bioMerieux, Marcy l’Etoile, France), or E-tests (AB Biodisk, Mannheim, Germany) applying EUCAST guidelines. Presumptive ESBL-, plasmidal AmpC-, or carbapenemase-producing isolates were analysed for presence of resistance genes by a DNA-array (Check-MDR CT103, Check-points, Wageningen, The Netherlands).

If patients tested positive they were isolated during their stay in the hospital, according to national and local guidelines. All patients diagnosed with TB or some patients suspected to have TB are referred directly to the UMCG tuberculosis centre and sanatorium Beatrixoord. This facility serves as one of the two national referral centre’s for tuberculosis and is the largest designated tuberculosis centre in Europe. This study was evaluated by the ethics committee and was waived in accordance with Dutch legislation owing to its retrospective nature (University Medical Centre Groningen, METc number 2014/325). No written informed consent was obtained from patients for the use of retrospective data but patient information was anonymized and de-identified prior to analysis.

### Statistical Analysis

Data was collected in and analyzed with SPSS (version 2.22) and descriptive statistics were used for the diagnosis and MDRO screening results. Data is presented as mean (SD) or median with 25–75% inter quartile range (IQR) as appropriate. General data was obtained from the patients’ file. When the date of arrival was only mentioned the year, either the 1^st^ July or 1^st^ of January was entered based on the information available.

The association between the number of days in the Netherlands and performing a screening for MDRO was calculated by Mann-Whitney-U test.

## Results

### Study Population and Group Characteristics

Between April 1st 2014 through September 1st 2015 care was provided to 736 asylum seekers in our university hospital. We included 273 patients who presented at the emergency ward or were admitted to the ward for further analysis. General characteristics can be found in Table 1. Most people originated from Eritrea (36.5%) or Syria (18.6%). Thirty-three (12%) asylum seekers were babies born in the Netherlands.

**Table 1:** General characteristics of asylum seekers admitted or presenting at the emergency department.

	Number of asylum seekers
**Male (%)**	184 (67%)
Days in the Netherlands Median (IQR)*	74 (22–247)
**Age median (IQR)**	24 (15–33)
**Country of origin**	
**Eritrea (%)**	92 (36.5)
**Syria (%)**	47 (18.6)
**Afghanistan (%)**	8 (3.2)
**Armenia (%)**	21 (8.3)
**Nigeria (%)**	7 (2.8)
**Other, Africa (%)**	38 (15.1)
**Other, Asia (%)**	21 (8.3)
**Other, Middle East (%)**	12 (4.8)
**Other, Europe (%)**	5 (2)
**Other, South America (%)**	1 (0.4)
**Missing (%)**	21 (8.3)

*In 153 patients, arrival date in the Netherlands had not been recorded

The median number of days in the Netherlands before presentation in the hospital was 74 (IQR 24–283). Many of the patients (32%) were admitted to the hospital or presented to the emergency department within the first 4 weeks after arrival in the Netherlands. Fourteen patients were admitted within the first week of arrival: 10 of whom were admitted within the first three days after arrival. In 56% (n = 153) no arrival date was reported in the patient documentation. Patients were admitted for a median duration of 7 (IQR2-26) days.

### Purpose of Hospital Visit

130 patients were admitted with an infectious disease of which 23% presented with vivax malaria (n = 30) and 34% proved to have pulmonary tuberculosis (n = 44). Three patients with pulmonary tuberculosis had drug resistant tuberculosis: one patient with MDRTB (from Georgia), one patient with XDRTB (from Latvia) and one patient with INH resistant pulmonary tuberculosis who originates from Syria but lived in both the Ukraine and Libya before seeking asylum in the Netherlands. 186 patients presented with non-infectious disease, with 22% (n = 40) associated with pregnancy, childbirth and post-partum care, 11% (n = 22) with diseases of the circulatory system and 12% with injury, poisoning and other consequences of external cause (n = 21). The diseases asylum seekers presented with are described in detail in Table 2. Eleven admitted patients were coinfected with HIV.

**Table 2:** Purpose of visit; infectious and non-infectious diseases.

**Infectious diseases**	**Number (%)**
Bacterial; pulmonary tuberculosis (n = 44), suspected tuberculosis (n = 9) intestinal tuberculosis (n = 1), tuberculous peritonitis (n = 1), relapsing fever *Borrelia recurrentis* (n = 2)	57 (43.8)
Parasitic; malaria (*P*. *vivax* n = 28, *P*. *falciparum* n = 2), leishmaniasis (n = 1), schistosomiasis (n = 2), (scabies n = 7)	40 (30.8)
Clinical presentation of an infection, not otherwise specified; fever, diarrhoea, abscess, respiratory infection, perinatal infection, deep infection of the finger, viral infection, tonsillitis, gastroenteritis, pharyngitis, eosinophilia	17 (13.1)
Viral; viral bronchiolitis (n = 2), viral respiratory infection (n = 1), hepatitis C (n = 9), cytomegalovirus (n = 1), disseminated Varicella Zoster Virus infection (n = 2)	15 (11.5)
Fungus; nasopharyngeal candida	1 (0.8)
**Total**	**130 (100)**
**Non infectious diseases**	**Number (%)**
pregnancy, childbirth and the puerperium	40(21.5)
injury, poisoning and certain other consequences of external causes	23(12.4)
diseases of the circulatory system	22(11.8)
certain conditions originating the perinatal period	19(10.2)
genitourinary system	14(7.5)
diseases of the nervous system	12(6.4)
endocrine, nutritional and metabolic diseases	9(4.8)
external causes of morbidity and mortality	8(4.3)
diseases of the musculoskeletal system and connective tissue	5(2.7)
diseases of the digestive system	5(2.7)
diseases of the blood and immune system	5(2.7)
diseases of the eye and adnexa	4(2.1)
diseases of the ear and mastoid process	4(2.1)
congenital malformations, deformations and chromosomal abnormalities	4(2.1)
Neoplasms	4 (2.1)
mental and behavioural disorders	4(2.1)
diseases of the respiratory system	3(1.6)
diseases of the skin and subcutaneous tissue	3(1.6)
**Total**	**186 (100.0)**

### Multi Drug Resistant Organisms

Of the 130 patients tested, 31% (n = 40) had one or more MDRO cultured, in total 52 MDROs. ESBL expressing *E*. *coli* (n = 20) was the most common MDRO. Additionally four *K*. *pneumoniae* and one *M*. *morganii* and one *E*. *cloacae* were found ESBL positive. Thirteen from the 26 ESBL positive Enterobacteriaceae were resistant to fluoroquinolones and at least one of the aminoglycosides (both tobramycin and gentamicin were tested). Genes encoding for CTX-M-1-like, CTX-M-15-like, and CTX-M-9 group ESBLs were detected in 13 (50%), 6 (23%), and 5 (19%) isolates, respectively. SHV 238S/240K was detected in one isolate. In one isolate with ESBL phenotype no resistance genes were detected by DNA array. Sixteen Enterobacteriaceae (mainly *E*. *coli*) were resistant to aminoglycosides and fluoroquinolones without ESBL. One *E*. *coli* isolate was resistant to colistin. No carbapenemase-producing Enterobacteriaceae were found. With respect to gram positive MDROs, only ten patients were found to carry MRSA.

MDRO carriage appeared to be higher among people from Syria than from Eritrea (7/13 vs 14/64, RR 2.46 (95% CI: 1.24–4.88)). Carriage of a MDRO was significantly associated with a shorter duration of stay in the Netherlands: median days in the Netherlands of those with MDRO was 26 (IQR: 4–87) days versus those without MDRO 85 (IQR: 27–316) days, P<0.001. No MDRO was cultured in asylum seekers’ babies born in the Netherlands (n = 9).

## Discussion

Around half of the asylum seekers admitted at our university hospital presented with an infectious condition. The carriage rate of MDRO in asylum seekers was 31%. Carriage rate varied by the patients’ country of origin and the duration of stay in the Netherlands, however, no CPE was detected.

Given the number of asylum seekers presenting at the national registration centre in Ter Apel, up to 800 daily, the number of admitted patients or patients referred to the emergency department at our university medical centre was low considering the likely adverse conditions during transit.

The most common infectious diseases patients presented with in our hospital were tuberculosis and *P*. *vivax* malaria. An increase in *P*. *vivax* malaria in newly arrived Eritrean asylum seekers has been noticed before in Sweden and Norway and its increase seems related to the migration route [19].

The high number of tuberculosis patients in our study results both from a higher incidence in many countries of origin, from the screening by X-ray at arrival in the national reception centre in Ter Apel, and from the asylum seekers with tuberculosis referred by other hospitals in the Netherlands to the UMCG tuberculosis centre. Patients also presented with diseases that are less common such as leishmaniasis or even more seldomly diagnosed in Europe such as the LBRF. After the two patients who reported to our hospital with LBRF [20] additional patients were reported in Switzerland [21] and Germany [22]. Because of the short incubation period the infection is likely to present quickly after arrival and thus at hospitals near to the single national registration centre.

Knowledge about infectious diseases and carriage of MDRO’s in asylum seekers is urgently needed to provide adequate care and to enable optimal hospital hygiene strategies. The carriage of MDRO in asylum seekers is high when compared to the Dutch population and also correlates to the carriage rate in country of origin as expected. Asylum seekers have a carriage rate of resistant Enterobacteriaceae comparable to Dutch inhabitants travelling abroad who are similarly known to import multi-drug resistant pathogens. Travellers from the Netherlands showed a high carriage rate of 30.5% of extended-spectrum β-lactamase-producing Enterobacteriaceae (ESBL-E) after their return from Asia, Africa or South America [23]. It should be considered whether screening policies should not only focus on asylum seekers, but also to consider screening Dutch patients admitted after international travels to Asia, Africa or South America as well. As an important fact, no CPE was found in asylum seekers. This is different to expectation, as regions of surrounding the country of origin of the asylum seekers are reported to have high prevalence of CPE [24]. As the prevalence of CPE is rising in other European countries, especially in South Europe, but also Germany, asylum seekers that have been treated in hospitals in those countries might get colonized during their travel to the Netherlands. Screening activities needs to be enhanced in order to identify CPE-carriers early. A recent study from Germany showed CPE-carriers and found a multidrug-resistant Gram-negative bacteria carriage rate of almost 61% [25] which is much higher than the MDRO carriage rate in our study. A higher background rate of MDRO in Germany, differences in travel routes and origin of asylum seekers and morbidity on admission, may all have contributed to the difference in MDRO carriage rate.

The aim of this study was to identify and list infectious diseases and carriage of high-risk potential pathogens that may have consequences for public health and infection control. We did not describe details of the non-communicable diseases asylum seekers presented with even though we realize that treatment of these non-communicable diseases are challenging considering the need of optimal compliance and follow-up [26].

The selection of asylum seekers in our hospital based on the insurance number is practical and ensures a complete selection of study participants. Selection based on information in the medical files is likely to be incomplete and selection based on the patients’ address leads to exclusion of asylum seekers do not live in the asylum centres or who have been transferred to other centres. The geographical location close to the single national registration centre ensures a true reflection of infectious diseases entering the Netherlands, especially considering the short incubation period from some of the infectious diseases.

Only the diagnoses at admissions were included because of their immediate importance for hospital hygiene measures. Purpose of visits to the out-patient clinic was not reported in this study. Psychiatric disorders are common in asylum seekers [27]. In our study, only four patients were admitted due to psychiatric disorders. However, most likely this low number does not reflect the actual prevalence; Most frequently, in the Netherlands, these patients are referred to specialised regional units for transcultural psychiatry.

Another limitation to the study is the percentage of asylum seekers screened at admission. Screening of admitted asylum seekers or asylum seekers presenting at the emergency department was only partially implemented and as a result screening was only done in 48% of the patients. Additional screenings is needed to identify the risk factors for carriage of MDRO strains. These additional screenings will also provide more details on the antimicrobial resistance. Further typing of the MDRO may provide information on the likely route of transmission.

In conclusion, asylum seekers frequently present with infectious diseases, of which many have consequences for infection control. Hospital staff should be prepared to recognize uncommon, poverty-related infectious diseases, especially in hospitals seeing patients who have recently arrived in the Netherlands. A close collaboration with the municipal health centre’s and the general practitioners at the asylum centres enables a rapid response to new events. Screening for MDRO at admission is necessary at least for originating countries with a high background rate of MDRO to enable the optimal treatment for patients and optimal strategy for infection control.
